# A phase I pharmacokinetic and safety study of cabazitaxel in adult cancer patients with normal and impaired renal function

**DOI:** 10.1007/s00280-016-3175-7

**Published:** 2016-10-27

**Authors:** Analía Azaro, Jordi Rodón, Jean-Pascal Machiels, Sylvie Rottey, Silvia Damian, Richard Baird, Javier Garcia-Corbacho, Ron H. J. Mathijssen, Pierre-François Clot, Claudine Wack, Liji Shen, Maja J. A. de Jonge

**Affiliations:** 1 Molecular Therapeutics Research Unit, Department of Medical Oncology, Vall d’Hebron University Hospital, Barcelona, Spain; 2 Pharmacology Department, Universitat Autònoma de Barcelona (UAB), Barcelona, Spain; 3 Department of Medical Oncology, Institut Roi Albert II, Cliniques Universitaires Saint-Luc and Institut de Recherche Clinique et Expérimentale (Pole MIRO), Université Catholique de Louvain, Brussels, Belgium; 4 Department of Medical Oncology, University Hospital of Ghent and Heymans Institute of Pharmacology, Ghent University, Ghent, Belgium; 5 Department of Medical Oncology, Fondazione IRCCS National Cancer Institute of Milan, Milan, Italy; 6 Early Phase Clinical Trials Team, Department of Oncology, University of Cambridge, Cambridge, UK; 7 Erasmus MC Cancer Institute, Erasmus University Medical Center, Rotterdam, The Netherlands; 8 Sanofi, Chilly-Mazarin, France; 9 Sanofi, Bridgewater, NJ USA

**Keywords:** Cabazitaxel, Renal impairment, Pharmacokinetics, Phase I, Advanced solid tumors

## Abstract

**Purpose:**

Limited data are available on cabazitaxel pharmacokinetics in patients with renal impairment. This open-label, multicenter study assessed cabazitaxel in patients with advanced solid tumors and normal or impaired renal function.

**Methods:**

Cohorts A (normal renal function: creatinine clearance [CrCL] >80 mL/min/1.73 m^2^), B (moderate renal impairment: CrCL 30 to <50 mL/min/1.73 m^2^) and C (severe impairment: CrCL <30 mL/min/1.73 m^2^) received cabazitaxel 25 mg/m^2^ (A, B) or 20 mg/m^2^ (C, could be escalated to 25 mg/m^2^), once every 3 weeks. Pharmacokinetic parameters and cabazitaxel unbound fraction (*F*_U_) were assessed using linear regression and mixed models. Geometric mean (GM) and GM ratios (GMRs) were determined using mean CrCL intervals (moderate and severe renal impairment: 40 and 15 mL/min/1.73 m^2^) versus a control (90 mL/min/1.73 m^2^).

**Results:**

Overall, 25 patients received cabazitaxel (median cycles: 3 [range 1–20]; Cohort A: 5 [2–13]; Cohort B: 3 [1–15]; and Cohort C: 5 [1–20]), of which 24 were eligible for pharmacokinetic analysis (eight in each cohort). For moderate and severe renal impairment versus normal renal function, GMR estimates were: clearance normalized to body surface area (CL/BSA) 0.95 (90% CI 0.80–1.13) and 0.89 (0.61–1.32); area under the curve normalized to dose (AUC/dose) 1.06 (0.88–1.27) and 1.14 (0.76–1.71); and *F*_U_ 0.99 (0.94–1.04) and 0.97 (0.87–1.09), respectively. Estimated slopes of linear regression of log parameters versus log CrCL (renal impairment) were: CL/BSA 0.06 (−0.15 to 0.28); AUC/dose −0.07 (−0.30 to 0.16); and *F*_U_ 0.02 (−0.05 to 0.08). Cabazitaxel safety profile was consistent with previous reports.

**Conclusions:**

Renal impairment had no clinically meaningful effect on cabazitaxel pharmacokinetics.

## Introduction

Impaired renal function is often observed in patients with solid tumors, with 40–60% reporting abnormal or impaired renal function across several studies [1–8]. Renal impairment can be the result of advanced age or chronic comorbidities, such as diabetes, hypertension or kidney disease, or can be caused by the cancer itself or the cancer treatment received [1, 8–13]. Several cancer therapies are nephrotoxic, including some chemotherapies, targeted agents, analgesics, radiopharmaceuticals, radiology contrast agents and antiresorptive agents [1]. For some anticancer therapies, including cisplatin, renal impairment is a contraindication; for example, 40–50% of patients with advanced bladder cancer cannot receive cisplatin because of its associated nephrotoxicity [8]. Renal impairment in patients receiving treatment for cancer is often associated with diminished drug metabolism and metabolite excretion, along with changes in absorption, renal and hepatic metabolism, and plasma protein binding and distribution, which all lead to altered pharmacokinetics (PK) of the drug received [11–13].

In the Renal Insufficiency and Anticancer Medications (IRMA) study, of 4684 patients with solid tumors (breast, colorectal cancer, lung, ovarian and prostate cancer), 57 and 53% of patients had renal impairment, depending on the type of formula used to calculate renal function, and of the 222 patients with prostate cancer, 63 and 56% of patients presented with renal impairment [2, 5]. Cabazitaxel is a second-generation taxane that has demonstrated efficacy in the second-line treatment of castration-resistant prostate cancer after docetaxel-based treatment [14]. Although renal elimination of cabazitaxel is minimal (approximately 2.3% is excreted renally as unchanged drug) [10, 15, 16], previous studies of cabazitaxel in patients with solid tumors have generally excluded patients with renal impairment [14, 17]. Some data on the PK of cabazitaxel in patients with renal impairment are available from a population PK analysis of phase I–III trials [16]. Of 170 patients included in this analysis, 59 patients had mild renal impairment, 14 patients had moderate renal impairment, and one patient had severe renal impairment. As expected with the minimal renal elimination of cabazitaxel, the population PK analysis did not identify renal impairment as a significant covariate influencing cabazitaxel PK.

The present study was performed to confirm the results of the previous population PK analysis [16] and to provide guidance regarding cabazitaxel dosing in patients with renal impairment. This study assessed the PK and safety of cabazitaxel in patients with advanced solid tumors and moderate or severe renal impairment compared with normal renal function. The primary objective of this study was to assess the effect of moderate and severe renal impairment on the PK of cabazitaxel. The secondary objective was to assess the safety of cabazitaxel in patients with moderate and severe renal impairment.

## Materials and methods

### Study design

This was an open-label, multicenter, phase I study (NCT01527929) in patients with advanced solid tumors and varying degrees of stable, chronic renal impairment or normal renal function. Patients were enrolled into one of three cohorts at seven institutions across five countries. Renal function cohorts were defined by creatinine clearance (CrCL), calculated using the Chronic Kidney Disease Epidemiology Collaboration equation (CKD-EPI) formula [18–20]. Patients were enrolled into Cohort A: normal renal function (CrCL >80 mL/min/1.73 m^2^), Cohort B: moderate renal impairment (CrCL ≥30 to <50 mL/min/1.73 m^2^) or Cohort C: severe renal impairment (CrCL <30 mL/min/1.73 m^2^).

Cabazitaxel was provided as a sterile non-pyrogenic solution in 60-mg vials, diluted into a premix solution prior to use and administered within 8 h of preparation. All patients received 1-h intravenous (IV) infusions of cabazitaxel on Day 1 of 3-weekly cycles until unacceptable toxicity, disease progression, withdrawal of consent, investigator decision or study cutoff. Cabazitaxel starting doses were based on the renal function stratification. Based on available PK data for patients with normal renal function and moderate renal impairment, no significant changes of cabazitaxel PK were expected in these patients. Therefore, the approved cabazitaxel dose of 25 mg/m^2^ was administered to patients with normal renal function (Cohort A) or moderate renal impairment (Cohort B). For patients with severe renal impairment, cabazitaxel starting dose was 20 mg/m^2^, escalated to 25 mg/m^2^ at later cycles if no dose-limiting toxicities (DLTs) were observed during Cycle 1. For Cohort C, PK assessment was carried out at the two dose levels for patients who received 25 mg/m^2^ at later cycles. The 20 mg/m^2^ starting dose was based on a possible increase in the free fraction of cabazitaxel in patients with severe renal impairment due to its high plasma protein binding (91.6%, mostly to albumin and lipoproteins) and the frequency of hypoalbuminemia in such patients. DLTs were defined as the following cabazitaxel-related adverse events (AEs, as assessed by the investigator): grade 2 vomiting and/or diarrhea; grade 3–4 non-hematologic AE (excluding grade 3 fatigue and transaminase or bilirubin elevation that returned to baseline prior to next treatment cycle); hematologic toxicity, defined as neutropenic infection, febrile neutropenia (fever of unknown origin without documented infection, with grade 3–4 neutropenia), grade 4 neutropenia lasting >7 days, or grade 3–4 thrombocytopenia. Toxicity and AEs were graded and recorded according to National Cancer Institute Common Terminology Criteria for AEs (NCI CTCAE) v4.03 [21]. Granulocyte-colony stimulating factor (G-CSF) was allowed with therapeutic or prophylactic intent and left to the investigator's judgement.

### Patient population

Patients had a diagnosis of a histologically or cytologically proven non-hematologic malignancy that was refractory to standard therapy or for which no standard therapy was available, and for which cabazitaxel was judged to be an adequate treatment option by the investigator. Eligible patients were ≥18 years of age with a life expectancy of >3 months, an Eastern Cooperative Oncology Group (ECOG) performance status (PS) of 0, 1 or 2, stable renal function (defined as CrCL within a range of ±10% during a 3-month period with ≥3 measurements performed), and adequate liver and bone marrow function (defined as absolute neutrophil count ≥1.5 × 10^9^/L, platelets ≥100 × 10^9^/L, total bilirubin ≤1.0 × institution upper limit of normality [ULN], transaminases ≤2.5 × ULN, and alkaline phosphatase ≤2.5 × ULN). Patients must have completed prior anticancer therapy ≥4 weeks before study entry. Key exclusion criteria included neurotoxicity of grade ≥2, acute renal failure or dialysis that would be required during the study, history of hypersensitivity to docetaxel or polysorbate 80, known brain metastases, and any treatments known to strongly induce CYP3A isoenzymes or to strongly inhibit CYP3A4 activity within 2 weeks before or during the test period for PK sampling. The institutional review board at each participating institution approved the protocol. Written informed consent was obtained from all patients. The study was conducted in accordance with the ethical principles stated in the Declaration of Helsinki and good clinical practice.

### Baseline and on study assessments

Medical histories were recorded at baseline and assessment of vital signs, physical examinations, ECOG PS, and electrocardiograms were performed at baseline prior to cabazitaxel administration, during study treatment as required and ≥30 days after the last administration of study treatment. All signs and symptoms observed from the time of informed consent were recorded as AEs. All AEs were recorded until 30 days after last administration of study treatment. After this follow-up period, only new or ongoing treatment-related AEs were recorded, except for ongoing serious AEs, which were assessed until resolution or stabilization, regardless of relationship to study treatment. Weekly laboratory evaluations were performed including cell blood counts with differentials, and analysis of coagulation, liver function, plasma electrolytes, glucose, albumin, total proteins, blood urea nitrogen, creatinine and urinalysis. An eye examination was performed at baseline and end of study. CrCL was determined using the CKD-EPI formula and if CrCL decreased by 50% from baseline during the study, or if dialysis was required, treatment was discontinued. The maximum dose delay allowed, due to acute toxicity, was 2 weeks. If the treatment gap was longer than 2 weeks, the patient was discontinued from study treatment. If the dose was reduced due to toxicity, then it was not re-escalated. Up to a maximum of two dose reductions were allowed per patient. Radiologic studies for disease assessment were conducted pretreatment and according to the site practice. Tumor response was assessed according to the Response Evaluation Criteria in Solid Tumors (RECIST 1.1).

### Pharmacokinetic sampling and bioanalytical methodology

Heparinized blood samples were collected from all patients for cabazitaxel concentration measurement at Cycle 1 (and Cycle 2, or Cycle 3 for one patient, following dose escalation in Cohort C) before start of infusion, 5 min before the end of infusion and then 5, 15 and 30 min, and 1, 2, 3, 5 and 8 h post-infusion, and at approximately 24 (Day 2), 48 (Day 3), 72 (Day 4), 120 (Day 6), 168 (Day 8) and 216 (Day 10) h after the end of infusion. The timing of treatment administration and timing of sampling were precisely recorded for each patient. Total cabazitaxel concentrations in the plasma were determined using a validated liquid chromatography with tandem mass spectrometry method (LC–MS/MS) with a lower limit of quantification (LLOQ) of 1 ng/mL [22, 23]. In addition, blood samples were collected to determine the free, unbound fraction of cabazitaxel in all patients at Cycle 1 before start of infusion, 5 min before the end of infusion, and 3 and 24 h after end of infusion. Free cabazitaxel concentrations were determined after equilibrium dialysis in buffer using a validated LC–MS/MS method with a LLOQ of 0.1 ng/mL.

### Pharmacokinetic endpoints and analysis

The primary PK endpoints were area under the plasma concentration versus time curve (AUC) and cabazitaxel clearance (CL). Secondary PK endpoints included observed maximum plasma concentration (*C*_max_), volume of distribution at steady state (*V*_ss_) and elimination half-life (*t*_1/2*ʎ*3_). *V*_ss_ and CL normalized by body surface area (BSA; *V*_ss_/BSA and CL/BSA) were also calculated. Total plasma concentrations of cabazitaxel and relative actual time values (as well as actual dose) were used to calculate the PK parameters using non-compartmental analysis (for *C*_max_) and individual modeling using a three-compartment open model with first-order elimination (for CL, AUC, *V*_ss_ and *t*_1/2*ʎ*3_). The calculation of PK parameters was performed using validated softwares (PKDMS version 2 running with WinNonlin Professional, version 5.2.1, Pharsight and WinNonlin Professional, version 6.3, Phoenix, Pharsight), as described previously [24, 25].

### Statistical analysis

Sample size for this study was based on empirical considerations and on the experiences in previous population studies; no formal sample size calculation was performed. A total of 24 to 27 patients were expected to be enrolled (at least 8 patients enrolled and evaluable for final PK evaluation in each cohort). One patient with a major deviation (missing PK sample at a critical time point) was excluded from the PK analysis. Statistical analysis evaluated the effect of population group on cabazitaxel PK parameters by modeling the relationship between measures of renal function (CrCL) and the PK parameters with a regression model as recommended by the United States Food and Drug Administration (FDA) and European Medicines Agency (EMA) [12, 13]. Log-transformed PK parameters were analyzed using a linear regression model with the independent variable being log-transformed CrCL at screening. Log-transformed BSA was a covariate in the models except in models using BSA normalized parameters. The effect of renal impairment on cabazitaxel PK parameters was analyzed using a linear fixed effects model. Geometric mean estimates were determined using log CrCL values corresponding to the mean boundaries of the CrCL interval covering the patient cohorts with moderate renal impairment (40 mL/min/1.73 m^2^) and severe renal impairment (15 mL/min/1.73 m^2^), along with a value representing the normal population and defined as the control (90 mL/min/1.73 m^2^). Using the regression model parameter estimates, point estimates for PK parameters corresponding to CrCL of 90, 40 and 15 mL/min/1.73 m^2^ were calculated after converting these values to the log scale. Geometric mean estimates were computed, and estimates for the geometric mean ratio of each population group (40 and 15 mL/min/1.73 m^2^) versus the control population group (90 mL/min/1.73 m^2^) were calculated. A similar analysis was performed for the models using BSA normalized parameters except log BSA was not used. Cabazitaxel unbound fraction was measured longitudinally in each patient. To estimate cabazitaxel unbound fraction, a linear mixed model was used with log BSA, log CrCL at screening and time as fixed effects, and random intercept and time slope as random effects.

## Results

### Patient characteristics

**Fig. 1:**
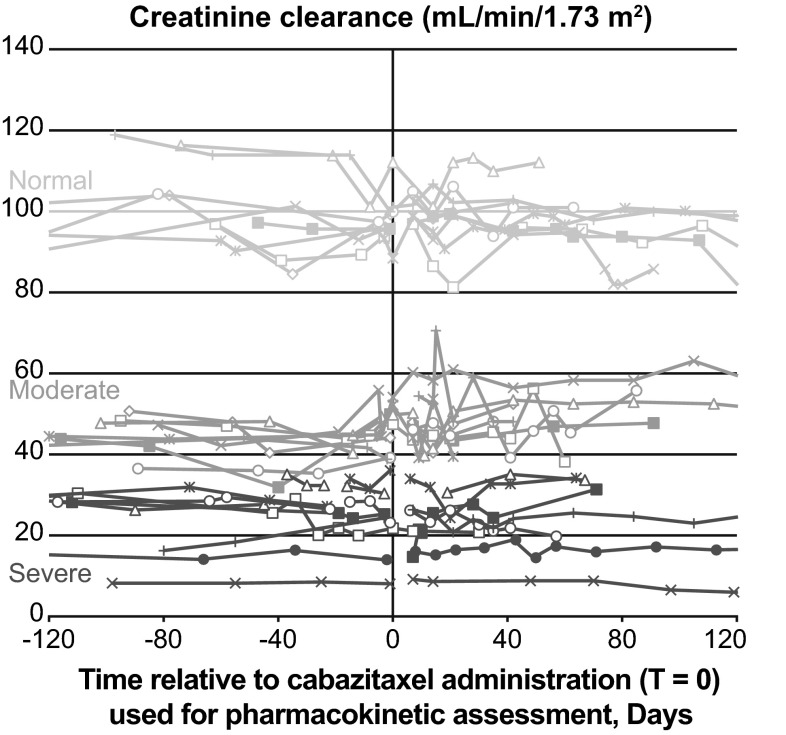
Creatinine clearance levels observed during the study period; an example observation window of 120 days before to 120 days after cabazitaxel administration and pharmacokinetic assessment is presented

Of 32 patients screened, 25 were enrolled between April 2012 and November 2013 including eight in Cohort A (normal renal function), eight in Cohort B (moderate renal impairment) and nine in Cohort C (severe renal impairment; Table 1). No patients failed screening due to renal function. All 25 patients were Caucasian and there was a balanced male/female distribution across all cohorts (Table 1). Baseline liver function appeared consistent across the cohorts with similar rates of hypoalbuminemia (Cohort A: 0; Cohort B: 1/8; Cohort C: 1/9), increased alkaline phosphatase levels (Cohort A: 4/8; Cohort B: 3/8; Cohort C: 2/9), and increased aspartate aminotransferase levels (Cohort A: 0; Cohorts B: 1/8; Cohort C: 1/9), reported at baseline for the three cohorts. Most patients (60%) had received ≥3 prior anticancer therapies, prior surgery had been performed in 80%, and prior radiation therapy had been administered to 48% of patients. Median CrCL at baseline was 96.8 mL/min in patients with normal renal function (Cohort A), 44.6 mL/min in patients with moderate renal impairment (Cohort B) and 25.2 mL/min in patients with severe renal impairment (Cohort C). The CrCL remained stable in all patients during the study period (Fig. 1).

**Table 1:** Baseline patient characteristics and treatment characteristics

	Cohort A (normal renal function) 25 mg/m^2^ *n* = 8	Cohort B (moderate renal impairment) 25 mg/m^2^ *n* = 8	Cohort C (severe renal impairment)			All patients *N* = 25
			All patients (starting dose 20 mg/m^2^) *n* = 9	Received only 20 mg/m^2^ *n* = 5^a^	Escalated to 25 mg/m^2^ *n* = 4	
Patient characteristics						
Male/female, *n*	2/6	4/4	5/4	3/2	2/2	11/14
Age, years, median (range)	58.5 (38–72)	65.0 (42–77)	66.0 (44–77)	69.0 (61–77)	63.0 (44–75)	62.0 (38–77)
ECOG performance status, *n*						
0	5	1	2	2	0	8
1	3	6	7	3	4	16
2	0	1	0	0	0	1
Months since diagnosis, median (range)	51.8 (6.6–113.4)	37.2 (8.6–460.7)	44.0 (15.5–154.7)	84.6 (15.5–154.7)	41.0 (25.4–55.6)	44.0 (6.6–460.7)
Primary tumor site, *n*						
Breast	2	0	0	0	0	2
Cervix	0	2	1	0	1	3
Colon/rectum	3	0	2	1	1	5
Esophagus	0	2	0	0	0	2
Ovary	1	1	0	0	0	2
Pancreas	1	1	0	0	0	2
Prostate	0	0	2	1	1	2
Other^b^	1	2	4	3	1	7
Tumor histology, *n*						
Adenocarcinoma	6	3	5	2	3	14
Carcinoma	1	4	1	1	0	6
Sarcoma	0	1	1	1	0	2
Other	1	0	2	1	1	3
Extent of disease at study entry, *n*						
Locally advanced	0	0	1	1	0	1
Metastatic	8	8	8	4	4	24
Prior anticancer regimens, *n*						
1	1	1	3	2	1	5
2	1	3	1	1	0	5
≥3	6	4	5	2	3	15
Prior taxane therapy, *n*	4	4	3	0	3	11
Creatinine clearance, mL/min, median (range)	96.78 (93.3−101.1)	44.60 (38.8−49.9)	25.24 (8.0−29.0)	15.06 (8.0−25.2)	27.40 (26.5−29.0)	44.39 (8.0−101.1)
Treatment characteristics						
Cabazitaxel cycles, *n*						
Total	45.0	41.0	54.0	39.0	15.0	140.0
Median per patient (range)	5.0 (2–13)	3.0 (1–15)	5.0 (1–20)	6.0 (1–20)	4.0 (2–5)	3.0 (1–20)
Relative dose intensity, mg/m^2^/week, median (range)	91.6 (79.2–99.8)	99.7 (70.2–101.1)	99.0 (88.8–99.9)	99.3 (92.3–99.9)	98.6 (88.8–99.9)	98.3 (70.2–101.1)
Duration of study treatment, weeks, median (range)	15.1 (6.0–46.0)	9.0 (3.0–52.1)	15.1 (3.0–65.3)	18.0 (3.0–65.3)	12.1 (6.7–15.3)	11.0 (3.0–65.3)
Discontinued treatment, *n* (%)	8 (100)	8 (100)	9 (100)	5 (100)	4 (100)	25 (100)
Adverse event	0	2 (25.0)	1 (11.1)	1 (20.0)	0	3 (12.0)
Poor compliance to protocol	0	0	0	0	0	0
Disease progression	7 (87.5)	4 (50.0)	4 (44.4)	0	4 (100)	15 (60.0)
Lost to follow-up	0	0	0	0	0	0
Patient request	0	1 (12.5)	1 (11.1)	1 (20.0)	0	2 (8.0)
Other reason	1 (12.5)	1 (12.5)	3 (33.3)	3 (60.0)	0	5 (20.0)

^a^In Cohort C, one patient had a cabazitaxel dose reduction to 15 mg/m^2^ at Cycle 2; this patient received a 20 mg/m^2^ dose at Cycle 1 and was therefore included in the PK population and assessed at Cycle 1

^b^One patient each with the following primary tumor sites: skin (Cohort A), adrenal gland (Cohort B), lung (Cohort B), bladder (Cohort C, 20 mg/m^2^), muscle/soft tissue (Cohort C, 20 mg/m^2^), peritoneum (Cohort C, 20 mg/m^2^) and uterus (Cohort C, 25 mg/m^2^)

*ECOG* Eastern Cooperative Oncology Group

### Treatment characteristics

A total of 140 cycles of cabazitaxel were delivered to 25 patients. The median number of cabazitaxel cycles administered was three (range 1–20; Table 1). The median number of cabazitaxel cycles was similar for patients with varying degrees of renal function, and median relative dose intensity was >90% in all cohorts. The median duration of study treatment was also similar between cohorts (Table 1). More than one cycle of cabazitaxel 25 mg/m^2^ was received by all eight patients with normal renal function (Cohort A) and seven of eight patients with moderate renal impairment (Cohort B). The remaining patient in Cohort B received cabazitaxel 25 mg/m^2^ in Cycle 1 only and then received 20 mg/m^2^ in Cycle 2, following dose reduction due to febrile neutropenia. Of the nine patients with severe renal impairment in Cohort C who received a cabazitaxel starting dose of 20 mg/m^2^, four patients had a dose escalation to 25 mg/m^2^ (three patients at Cycle 2 and one patient at Cycle 3). The remaining five patients did not receive dose escalations because of DLTs (*n* = 3) and investigator decision (*n* = 2). In Cohort C, one DLT of grade 3 febrile neutropenia led to dose reduction to 15 mg/m^2^ at Cycle 2. No patient received two reductions of cabazitaxel dose, and no patient had dose interruptions.

### Pharmacokinetics

**Fig. 2:**
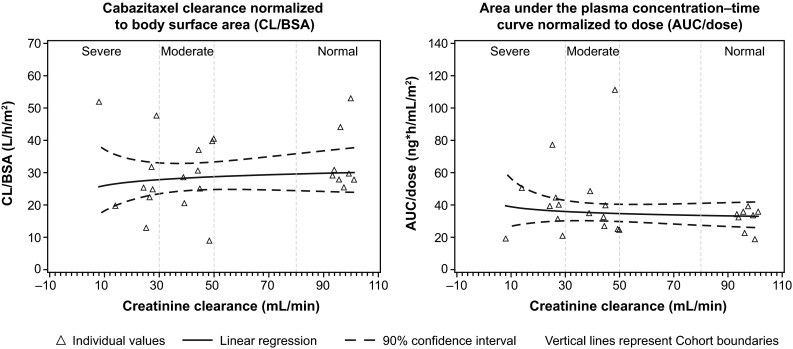
Estimated slope of linear regression for log pharmacokinetic parameters versus creatinine clearance

Cabazitaxel PK data were obtained from 25 treated patients and 24 were eligible for PK analysis (eight per cohort). One patient (Cohort C) was excluded due to protocol deviation. PK parameters obtained using non-compartmental analysis and three-compartmental analysis (individual modeling) are shown in Table 2. For patients with moderate or severe renal impairment (versus patients with normal renal function), there appeared to be no associations between CL/BSA or AUC normalized to dose (AUC/dose), and degree of renal impairment. Mean CL/BSA observed was similar across all patients with normal renal function (33.5 L/h/m^2^; geometric mean 32.5 L/h/m^2^), moderate renal impairment (28.9 L/h/m^2^; geometric mean 26.5 L/h/m^2^) and severe renal impairment (29.6 L/h/m^2^ for patients receiving 25 and 20 mg/m^2^ combined; geometric mean 27.0 L/h/m^2^). The effect of renal impairment on cabazitaxel PK parameters was evaluated in patients with moderate and severe renal impairment in comparison with patients with normal renal function using a regression model of patient PK parameters from the PK population using CrCL values obtained at screening. The primary linear regression of log-transformed parameters (AUC/dose and CL/BSA) versus log-transformed CrCL is shown in Fig. 2. Estimated slopes of linear regression for log PK parameters versus log CrCL were 0.06 (90% CI −0.15, 0.28) for CL/BSA and −0.07 (90% CI −0.30, 0.16) for AUC/dose. For primary PK parameters, linear regression analysis showed no meaningful increase in cabazitaxel dose-normalized exposure (AUC/dose: *p* = 0.5961) and no meaningful decrease in cabazitaxel CL/BSA (*p* = 0.6268) associated with the decrease in CrCL (increased renal impairment) within the range of 8.03–101 mL/min.

**Table 2:** Pharmacokinetic parameters

Parameter, mean ± SD (geometric mean) [CV %]	Cohort A (normal renal function) 25 mg/m^2^ *n* = 8	Cohort B (moderate renal impairment) 25 mg/m^2^ *n* = 8	Cohort C (severe renal impairment)	
			20 mg/m^2^ *n* = 4^a^	25 mg/m^2^ *n* = 4^b^
Non-compartmental analysis				
*C*_max_, ng/mL	161 ± 57.0 (152) [35]	241 ± 207^c^ (193) [86]	135 ± 45.7 (130) [34]	244 ± 150 (215) [62]
Individual modeling/three-compartmental analysis				
AUC, ng*h/mL	787 ± 177 (766) [23]	1070 ± 733 (938) [68]	928 ± 475 (829) [51]	857 ± 263 (823) [31]
CL, L/h	58.9 ± 14.7 (57.5) [25]	54.1 ± 21.9 (49.1) [41]	51.8 ± 34.4 (44.8) [66]	63.0 ± 30.5 (58.1) [48]
*V*_ss_, L	7730 ± 3280 (7160) [42]	6730 ± 2970 (6170) [44]	5810 ± 1360 (5690) [23]	6470 ± 3790 (5680) [59]
CL/BSA, L/h/m^2^	33.5 ± 9.76 (32.5) [29]	28.9 ± 10.7 (26.5) [37]	27.5 ± 17.1 (24.1) [62]	31.7 ± 11.4 (30.3) [36]
*V*_ss_/BSA, L/m^2^	4230 ± 1360 (4040) [32]	3580 ± 1480 (3320) [41]	3130 ± 730 (3060) [23]	3380 ± 1920 (2970) [57]
*t*_1/2*γ*3_, h	122 ± 43.8 (116) [36]	143 ± 102 (124) [71]	133 ± 84.4 (113) [63]	115 ± 49.8 (103) [43]

^a^ *n* = 4; three patients were not included in the statistical analysis for Cycle 1 (20 mg/m^2^) because they were analyzed at Cycle 2 (25 mg/m^2^)

^b^ *n* = 4; one patient was excluded from statistical analyses because the cabazitaxel dose was decreased to 15 mg/m^2^

^c^ *n* = 7; one patient was excluded from the calculation of summary statistics because of a sampling time deviation at the end of infusion

*AUC* Area under the plasma concentration–time curve, *CL* clearance, *CL/BSA* clearance normalized to body surface area, *C*_*max*_ maximum plasma concentration, *CV* coefficient of variation, *SD* standard deviation, *t*_*1/2γ3*_ elimination half-life, *V*_*ss*_ volume of distribution at steady state, *V*_*ss*_ */BSA* volume of distribution at steady state normalized to body surface area

Estimates for the geometric mean ratios of PK parameters for the renal impairment groups (moderate and severe) versus an estimated control group were determined from the linear regression model (Table 3). Log CrCL values corresponding to the mean boundaries of the CrCL interval covering the patient cohorts with moderate renal impairment (40 mL/min/1.73 m^2^) and severe renal impairment (15 mL/min/1.73 m^2^) were used, versus a value representing the normal population and defined as the control group (90 mL/min/1.73 m^2^). The geometric mean ratio for AUC in patients with severe renal impairment versus normal renal function (1.14; 90% CI 0.76, 1.71) indicated no significant impact of renal impairment. For CL/BSA, the geometric mean ratio for patients with severe renal impairment versus patients with normal renal function was 0.89 (90% CI 0.61, 1.32). The predicted CL/BSA for patients with severe renal impairment or normal renal function was 26.66 and 29.81 L/h/m^2^, respectively, which accounts for a maximal decrease in cabazitaxel clearance of 10.6% and indicates no meaningful change in CL/BSA with increased renal impairment. Some secondary PK parameters appeared to have trend for association with decreasing renal function; in particular, *V*_ss_/BSA was approximately 2700 L/m^2^ for patients with severe renal impairment versus approximately 4000 L/m^2^ for patients with normal renal function. The log of the linear regression slope was 0.22 (90% CI 0.02, 0.41), indicating that this trend was marginally statistically significant. Results from a sensitivity analysis, where CrCL changes during the time course of the study were taken into account, showed similar findings.

**Table 3:** Estimated pharmacokinetic parameters for specified levels of renal function (CrCL)

	Normal renal function (CrCL 90 mL/min/1.73 m^2^)^a^	Moderate renal impairment (CrCL 40 mL/min/1.73 m^2^)^a^	Severe renal impairment (CrCL 15 mL/min/1.73 m^2^)^a^
Geometric mean estimate (90% CI)			
CL/BSA^b^, L/h/m^2^	29.81 (24.18–36.75)	28.34 (24.44–32.86)	26.66 (20.15–35.27)
AUC/dose^c^, ng*h/mL/mg/m^2^	33.23 (26.65–41.44)	35.21 (30.24–40.99)	37.75 (28.29–50.39)
*V*_ss_/BSA^b^, L/m^2^	3991.24 (3305.36–4819.44)	3345.54 (2927.74–3822.96)	2702.50 (2100.18–3477.57)
Cabazitaxel* F*_U_^b^, %	5.51 (5.08–5.96)	5.44 (5.13–5.76)	5.36 (4.95–5.80)
Geometric mean ratios versus normal renal function (90% CI)			
CL/BSA^b^, L/h/m^2^	–	0.95 (0.80–1.13)	0.89 (0.61–1.32)
AUC/dose^c^, ng*h/mL/mg/m^2^	–	1.06 (0.88–1.27)	1.14 (0.76–1.71)
*V*_ss_/BSA^b^, L/m^2^	–	0.84 (0.72–0.98)	0.68 (0.48–0.96)
Cabazitaxel*F*_U_^d^, %	–	0.99 (0.94–1.04)	0.97 (0.87–1.09)

^a^Specified CrCL values correspond to the mean boundaries of the CrCL interval covering the patient cohorts with moderate renal impairment (40 mL/min/1.73 m^2^) and severe renal impairment (15 mL/min/1.73 m^2^), versus a value representing the normal population and defined as the control group (90 mL/min/1.73 m^2^)

^b^Model is linear regression: log (CL/BSA) = log (CrCL) + Error

^c^Model is linear regression: log (AUC/dose) = log (CrCL) + log (BSA) + Error

^d^Model is linear mixed: log (fraction unbound) = log (CrCL) + log (BSA) + Time + (b0 + b1 × Time) + Error

*AUC/dose* Area under the plasma concentration–time curve normalized to dose, *CI* confidence interval, *CL/BSA* clearance normalized to body surface area, *CrCL* creatinine clearance, *F*_*U*_ unbound fraction

The estimated unbound fraction of cabazitaxel was low and consistent across the different renal function cohorts (5.36–5.51), indicating that renal impairment had no meaningful effect on the cabazitaxel unbound fraction. This was further supported by the primary linear regression analysis and subsequent estimated slope of linear regression (0.02; 90% CI −0.05, 0.08). Unbound drug PK analysis would therefore lead to the same conclusions as for total drug.

### Safety

All patients experienced at least one treatment-emergent AE (TEAE) of any grade. Twenty-three patients (92%) experienced a treatment-related TEAE, which was grade 3–4 in 12 patients (48%). A treatment-related serious TEAE occurred in eight patients (32%). There were no specific patterns of AEs associated with renal impairment. For the most frequently reported TEAEs, incidence rates were not notably different between patients with different levels of renal function. The most frequent treatment-related grade 3–4 TEAE was febrile neutropenia in six patients (24%), including one patient with normal renal function (Cohort A, 12.5%), three patients with moderate renal impairment (Cohort B, 37.5%) and two patients with severe renal impairment (Cohort C, 22.2%), followed by diarrhea in three patients (12%), comprising two patients with moderate renal impairment (Cohort B, 25%) and one patient with severe renal impairment (Cohort C, 11.1%; Table 4). The most frequent grade 3–4 hematologic TEAE based on laboratory assessments was neutropenia which was reported in 21 patients (84%) including seven patients from each cohort of patients (Cohort A, 87.5%; Cohort B, 87.5%; Cohort C, 77.8%), followed by leukopenia in 20 patients (80%), comprising seven patients with normal renal function (Cohort A, 87.5%), seven patients with moderate renal impairment (Cohort B, 87.5%) and six patients with severe renal impairment (Cohort C, 66.7%; Table 4). Grade 3–4 laboratory abnormalities, such as isolated electrolyte imbalances, were reported in a small number of patients and are anticipated in this population of advanced cancer patients with renal insufficiency and some with possible paraneoplastic syndromes (Table 4). There was no specific pattern of abnormality detected. Differences among cohorts in the number of patients presenting with creatinine increase were also anticipated and were directly related to their renal function status. No grade 3–4 TEAEs related to renal and urinary toxicity were observed in any patient (a detailed overview of TEAEs is displayed in Table 4).

**Table 4:** Summary of treatment-emergent adverse events (TEAEs) and laboratory abnormalities

Patients, *n* (%)	Cohort A (normal renal function) 25 mg/m^2^ *n* = 8	Cohort B (moderate renal impairment) 25 mg/m^2^ *n* = 8	Cohort C (severe renal impairment)			All patients *N* = 25
			All patients (starting dose 20 mg/m^2^) *n* = 9	Received only 20 mg/m^2^ *n* = 5^a^	Escalated to 25 mg/m^2^ *n* = 4	
Grade 3–4 TEAEs, *n* (%)	6 (75.0)	5 (62.5)	8 (88.9)	5 (100)	3 (75.0)	19 (76.0)
Grade 3–4 treatment-related TEAEs,* n* (%)						
Any	4 (50.0)	3 (37.5)	5 (55.6)	4 (80.0)	1 (25.0)	12 (48.0)
Diarrhea	0	2 (25.0)	1 (11.1)	1 (20.0)	0	3 (12.0)
Asthenia	1 (12.5)	0	0	0	0	1 (4.0)
Dizziness	0	0	1 (11.1)	0	1 (25.0)	2 (8.0)
Fatigue	0	1 (12.5)	0	0	0	1 (4.0)
Abdominal pain	0	1 (12.5)	0	0	0	1 (4.0)
Febrile neutropenia	1 (12.5)	3 (37.5)	2 (22.2)	1 (20.0)	1 (25.0)	6 (24.0)
Soft tissue infection	1 (12.5)	0	0	0	0	1 (4.0)
Grade 3–4 hematologic TEAEs of any causality^b^, *n* (%)						
Anemia	1 (12.5)	1 (12.5)	1 (11.1)	1 (20.0)	0	3 (12.0)
Leukopenia	7 (87.5)	7 (87.5)	6 (66.7)	3 (60.0)	3 (75.0)	20 (80.0)
Neutropenia	7 (87.5)	7 (87.5)	7 (77.8)	4 (80.0)	3 (75.0)	21 (84.0)
Lymphopenia	3 (37.5)	4 (50.0)	3 (33.3)	2 (40.0)	1 (25.0)	10 (40.0)
Grade 3–4 laboratory abnormalities of any causality, *n* (%)						
Alkaline phosphatase increased	1 (12.5)	0	0	0	0	1 (4.0)
Hypercalcemia	2 (25.0)	0	0	0	0	2 (8.0)
Creatinine increased	0	0	4 (44.4)	4 (80.0)	0	4 (16.0)
Hyperkalemia	0	0	1 (11.1)	1 (20.0)	0	1 (4.0)
Hypokalemia	0	0	1 (11.1)	0	1 (25.0)	1 (4.0)
Hypermagnesmia	0	0	2 (22.2)	1 (20.0)	1 (25.0)	2 (8.0)
Blood bilirubin increased	1 (12.5)	0	0	0	0	1 (4.0)

	All grades	Grade 3–4	All grades	Grade 3–4	All grades	Grade 3–4	All grades	Grade 3–4	All grades	Grade 3–4	All grades	Grade 3–4
Renal and urinary TEAEs of any causality, *n* (%)												
Any renal and urinary disorder	3 (37.5)	0	1 (12.5)	0	1 (11.1)	0	1 (20.0)	0	0	0	5 (20.0)	0
Dysuria	1 (12.5)	0	0	0	0	0	0	0	0	0	1 (4.0)	0
Renal colic	1 (12.5)	0	0	0	0	0	0	0	0	0	1 (4.0)	0
Non-infective cystitis	1 (12.5)	0	0	0	0	0	0	0	0	0	1 (4.0)	0
Hematuria	1 (12.5)	0	0	0	0	0	0	0	0	0	1 (4.0)	0
Acute renal failure	0	0	1 (12.5)	0	0	0	0	0	0	0	1 (4.0)	0
Urinary retention	0	0	0	0	1 (11.1)	0	1 (20.0)	0	0	0	1 (4.0)	0
Discontinuations due to TEAEs, *n* (%)												
Any TEAE leading to treatment discontinuation	0	0	2 (25.0)	2 (25.0)	1 (11.1)	1 (11.1)	1 (20.0)	1 (20.0)	0	0	3 (12.0)	3 (12.0)
Cholecystitis, infective	0	0	1 (12.5)	1 (12.5)	0	0	0	0	0	0	1 (4.0)	1 (4.0)
Colitis, ischemic	0	0	0	0	1 (11.1)	1 (11.1)	1 (20.0)	1 (20.0)	0	0	1 (4.0)	1 (4.0)
Diarrhea	0	0	1 (12.5)	1 (12.5)	0	0	0	0	0	0	1 (4.0)	1 (4.0)
Pneumonia	0	0	0	0	1 (11.1)	0	1 (20.0)	0	0	0	1 (4.0)	0

^a^In Cohort C, one patient had a cabazitaxel dose reduction to 15 mg/m^2^ at Cycle 2; this patient received a 20 mg/m^2^ dose at Cycle 1 and was therefore included in the PK population and assessed at Cycle 1

^b^Hematoloigc TEAEs based on laboratory abnormalities

Patients with severe renal impairment in Cohort C were assessed for DLTs during Cycle 1 as part of the dose escalation determination. Three patients in Cohort C experienced a DLT during Cycle 1, including grade 3 febrile neutropenia, grade 3 diarrhea and grade 3 neutropenia.

Three patients (12%) discontinued treatment because of a TEAE (Table 4). Of patients with moderate renal impairment (Cohort B), two patients discontinued cabazitaxel because of treatment-related diarrhea (*n* = 1) and a serious AE (cholecystitis; *n* = 1) considered unrelated to treatment. Of patients with severe renal impairment (Cohort C), one patient receiving cabazitaxel 20 mg/m^2^ discontinued treatment because of serious AEs (ischemic colitis and pneumonia) considered unrelated to treatment.

### Efficacy

Efficacy assessments were not an objective of this study, but tumor response by RECIST criteria was evaluated per the investigators usual practice. Partial response was seen in two patients (8%) including one patient in Cohort A with breast cancer and one patient in Cohort C with bladder cancer. Stable disease was reported in 11 patients (44%) and progressive disease in nine patients (36%). Three patients were not evaluable for tumor response or did not have measurable disease at baseline.

## Discussion and conclusions

Patients with renal or hepatic impairment are generally excluded from phase I clinical trials due to the challenges they present [26], therefore most cancer treatments are approved with limited information on their PK in these patient populations. PK and safety studies in patients with renal impairment may be requested by regulatory authorities at the time of treatment approval, although many studies are conducted post-approval. The primary goal of phase I PK studies in patients with impaired renal function is to determine whether the PK is altered to such an extent that the dosage should be adjusted from the established, approved dose. There are many previous and ongoing PK studies performed in cancer patients [26–32]. For example, oxaliplatin and imatinib PK studies concluded that, even though drug clearance may be decreased and exposure increased, treatments were well tolerated and no dose reduction was necessary in patients with mild-to-moderate renal impairment [27, 28]. For other treatments such as vinflunine, pemetrexed and eribulin, PK studies supported a dose reduction from the approved dose in patients with renal impairment [29, 30, 32].

Since cabazitaxel is only minimally excreted via the kidneys (3.7 with 2.3% excreted as unchanged drug) [15], it was considered unlikely that renal impairment would influence the PK of cabazitaxel. This article details the results of the first study conducted to assess the safety and PK of cabazitaxel in patients with moderate or severe renal impairment, as this population of patients have been excluded from prior studies of cabazitaxel or minimally represented. In a previous population PK analysis of cabazitaxel, patients with moderate or severe renal impairment accounted for less than 10% of the patient population (15/170) [16]. The current phase I PK and safety study confirms the findings of the previous PK population analysis that cabazitaxel dose modification is not required for patients with moderate or severe renal dysfunction as renal impairment had no meaningful effect on the PK of cabazitaxel. Increasing renal impairment did not result in any meaningful increase in cabazitaxel dose-normalized exposure or decrease in cabazitaxel CL/BSA. Cabazitaxel clearance was similar regardless of renal impairment and was within the range of values observed in previous studies in patients with advanced solid tumors [16, 24, 25]. In two phase I studies, the mean cabazitaxel clearance rates were high (27.3–44.7 L/h/m^2^) [24, 25]. In the population PK assessment of cabazitaxel in patients with advanced solid tumors from five different studies, including the two phase I studies (total: *n* = 170; ranging between *n* = 13 and *n* = 67), the mean clearance of cabazitaxel ranged from 12.1 to 34.5 L/h/m^2^ [16], which was lower than those values obtained from the individual modeling [24, 25]. The population PK analysis allowed for a better estimation of the PK parameters from the phase I studies as the impact of sample times was reduced. Renal impairment also had no meaningful effect on the unbound fraction of cabazitaxel, which is consistent with the high binding of cabazitaxel to total plasma proteins observed ex vivo and in vitro (89–92%) [16, 25, 33].

The trends in PK parameters associated with renal function were not considered clearly established for any parameter because of the large variability in parameters that was not well accounted for by the linear regression models, indicating a limitation of the methodology. This was demonstrated by the lack of precision (large confidence intervals) in the estimates of both geometric mean ratios and point parameters in patients with severe renal impairment. In addition, outlier values in different parameters were seen in different patients. Including a cohort of patients with mild renal impairment in this study would have allowed for assessments over a wider range of renal function and provided information for patients with CrCL levels falling into the ≥50 to ≤80 mL/min/1.73 m^2^ interval, which may have refined the linear regression models. Patients with mild renal impairment were not included because cabazitaxel is minimally excreted by the kidneys, meaning that assessment of cabazitaxel in patients with moderate or severe renal impairment would provide the most clinically relevant information. In our opinion, the findings in patients with moderate or severe renal impairment suggest that further studies in patients with mild renal impairment are not required. However, for studies of new agents where the PK of certain treatments may be affected by renal impairment, obtaining data from a patient population spanning a continuous range of CrCL levels and including patients with a complete series of renal function and impairment, may be an ideal approach.

The overall safety and AE profile of cabazitaxel observed in patients with normal or moderate renal impairment in this study was consistent with the known safety profile of cabazitaxel, and no new safety issues were identified. For the most frequently reported TEAEs, incidence rates were similar for patients with different levels of renal function. The low rate of AEs related to renal toxicity in patients with renal impairment suggests that renal function does not decrease during cabazitaxel treatment. The overall rate of grade 3–4 neutropenia in this study, based on laboratory assessments, was 84%. This is similar to the rate of neutropenia reported in the phase III TROPIC trial (94% all grades, 82% grade 3–4) [14]. The overall rate of grade 3–4 febrile neutropenia in this study was 24%, compared with 8% in TROPIC. This apparent difference could be due to the smaller number of patients, the heterogeneous patient population, or the more heavily pretreated patient population, in this phase I study compared with TROPIC.

Patients with mild-to-moderate renal impairment pose an increasingly frequent challenge for clinicians. Taking into consideration the results of this study and other studies assessing the PK and safety of treatments in patients with renal dysfunction, patients with renal impairment could be considered for entry into selected phase I studies of treatments with low renal clearance, where it is deemed unlikely that renal impairment would affect drug PK [26]. This will provide data for new agents in this patient population earlier and increase patient access to clinical trials and experimental treatments. Furthermore, results from such PK studies would inform regulatory authorities as to whether dose modifications should be recommended for certain patient subpopulations.

In summary, the data support the clinical recommendation that full doses of cabazitaxel (25 mg/m^2^) can be safely administered every 3 weeks to patients with mild-to-severe renal dysfunction.
